# Announcement: 10th NCI-EORTC Symposium on New Drugs in Cancer Therapy

**DOI:** 10.1155/MBD.1997.346

**Published:** 1997


### 10 th NCI-EORTC SYMPOSIUM ON NEW DRUGS IN CANCER THERAPY

### Amsterdam, The Netherlands, June 16-19, 1998

This second announcement is to inform you on the scientific and social program of the 10th NCI-EORTC Symposium on New Drugs in Cancer Therapy, to be held 16-19 June, 1998.
The series of NCI-EORTC Symposia is heading for the tenth edition in June 1998. Over the past 11 years, this series has built its reputation as one of the worlds best meetings and certainly as the most dedicated meeting in the field of new anticancer therapies.
This meeting will cover actual developments in the field of anticancer drug research, both at the preclinical and the clinical level. Many top speakers have already agreed to present a lecture on their field of research at the symposium. The current announcement provides the provisional program. The format remains largely unaltered compared to the previous meetings, although more workshops will be held since these were highly appreciated at the previous symposium. In addition, the Scientific Advisory Board will be reviewing the submitted abstracts very critically to even further improve the quality of the poster presentations.
This will be the sixth consecutive meeting in this series to be organized by the EORTC New Drug Development Office in Amsterdam. The increase to over 1,400 active participants on the occasion of the 9th NCI-EORTC Symposium in March 1996 has demonstrated that the importance of this meeting is now being recognized by many in academia, industry, and governmental agencies.
More than ever before it will be important to register as early as possible to prevent disappointment. The maximum number of attendees that can be hosted is almost reached already. There will also be more pressure on hotel accommodations this time due to the summer period, a popular time for tourists to visit Amsterdam.

### History of the series

The series of NCI-EORTC Symposia are a perfect reflection of the close collaboration between the NCI (US National Cancer Institute) and the EORTC (European Organization for Research and Treatment of Cancer) over the past decades. These two organizations are heavily involved in the discovery of potential new anticancer agents and their subsequent development through the various phases of preclinical and early clinical development.
A series of specialized symposia, held every 2-3 years since 1978, has been dedicated to this theme. The first four of these symposia were held in Brussels, Belgium. In 1984, after Dr. Pinedo had established the EORTC New Drug Development Office (NDDO) in Amsterdam, the symposium moved to Amsterdam.
All subsequent symposia were organized by the NDDO, with Dr. Pinedo as the Symposium President, starting with the 5th NCI-EORTC Symposium on New Drugs in Cancer Therapy in October 1986.

### Global forum

Since then, the symposium has seen a strong growth to over 1,400 participants from 35 countries at the last meeting in March 1996. This growth has been achieved not only by increased participation from European countries; there has been an even stronger increase in the number of participants from other continents, notably North-America and the Far-East. Thus, approximately one-third of the participants at the 9th symposium were non-European. So, the series has achieved a truly global scale and provides an excellent forum to everyone involved in the research and development of new anticancer agents which nowadays tends to occur in a worldwide setting.

### Scope of the symposium

The symposium series aims to provide a forum for presentation and discussion of recent results in the research and development of new anticancer agents in a broad sense, including compounds from natural or synthetic origin, biological agents, immunological treatments, gene therapy, and drugs designed to modify the action and/or the toxicity of existing anticancer agents. The symposium covers both preclinical research and (early) clinical development of such drugs, including methodologies employed in each of the research areas involved.

The scientific program focuses on state-of-the-art presentations by top speakers, and is organized according to the highest possible standards. The program is of interest to everyone with a scientific interest in new anticancer drugs, e.g. in academic research institutes, the pharmaceutical industry, and governmental or multinational research and regulatory agencies.

**Scientific program**

The scientific program will run from Tuesday, June 16, 1998, to Friday, June 19, 1998, late afternoon. Elements of the program will be: the Opening Ceremony, including the Presidential Address, the Michel Clavel Lecture, the NDDO Honorary Award Presentation, and several Keynote Lectures; plenary lecture sessions in the morning and the afternoon; poster sessions; and daily poster reviews as part of the afternoon plenary sessions. Due to positive reactions of participants of the previous symposium, the number of workshops, allowing in-depth discussion on specific topics, will be increased. Plenary sessions will be focused on particular areas of interest in preclinical and clinical anticancer drug research like:

**New mechanisms and cellular targets for anticancer agents**
**Anti-angiogenesis and anti-metastatic agents**
**New DNA-interactive agents (e.g. marine compounds)**
**Gene therapy**
**Novel delivery systems and prodrugs**
**Tubulin inhibitors: other mechanisms and combinations**
**Camptothecins: problems to overcome and future prospects**
**Apoptosis**

Further topics will be announced in the final program.

Special attention will be given to aspects of translational research, linking preclinical (e.g. molecular biology and genetics) data and clinical findings in a given area. The majority of presentations will be given by invited speakers who are experts in the field. In addition, there will be a limited number of slots for oral presentation of submitted papers which receive high ratings in the review process, and the topics of which fit in one of the sessions.

**Poster sessions** will be held on Wednesday, Thursday and Friday. Poster topics will cover all areas within the scope of the symposium. Accepted posters will be displayed with ample time for viewing and discussion during the extended lunch breaks. During the plenary sessions in the afternoon an expert will present his/her review of posters displayed.

**Topics for discussion in workshops**, under supervision of a panel of experts, are:

**Tumor vaccines**
**Combinatorial chemistry / diversity generation**
**Early clinical trials with radiosensitizers**
**New endpoints for Phase I studies with new therapeutics**
**Pharmacokinetics and pharmacodynamics in drug development**
**Computer assisted drug development and molecular modelling**

Detailed planning and additional topics for workshops will be announced in the final program.

**Speakers and chairmen** (provisional list)

J.H. Beijnen Amsterdam, Netherlands
Th.R. Boon Brussels, Belgium
M.C. Christian Bethesda, U.S.A.
S.T. Crooke Carlsbad, U.S.A.
J.A. Double Bradford, U.K.
I.J. Fidler Houston, U.S.A.
J. Folkman Boston, U.S.A.
M. Friedman Rockville, U.S.A
G. Giaccone Amsterdam, Netherlands
A. Hanauske München, Germany
A.L. Harris Oxford, U.K.
F. Hausheer San Antonio, U.S.A.
W.K. Hong Houston, U.S.A.
S.B. Horwitz New York, U.S.A
R. Jain Boston, U.S.A.
S.B. Kaye Glasgow, U.K.
S.J. Korsmeyer St-Louis, U.S.A.
A. Matter Basel, Switzerland
F. McCormick San Francisco, U.S.A.
C. Melief Leiden, Netherlands
J. Mendelsohn New York, U.S.A.
H. Newell Newcastle, U.K.
H.M. Pinedo Amsterdam, Netherlands
S. Rosenberg / M. Sznol Bethesda, U.S.A.
J. Roth Houston, U.S.A.
N. Saijo Tokyo, Japan
E.A. Sausville Bethesda, U.S.A.
T. Tursz Villejuif, France
J. Verweij Rotterdam, Netherlands
D.D. Von Hoff San Antonio, U.S.A.
R.E. Wittes Bethesda, U.S.A.

Accreditation and certification

CME accreditation for the scientific program on behalf of medical oncologists participating in the scientific program is already approved by the European Society of Medical Oncology (ESMO) and is pending for the USA. Upon request, registered symposium participants will receive a certificate of attendance at the end of the symposium.

**Important dates**

**February 1, 1998: Deadline early registration**
**February 20, 1998: Deadline abstract submission**
**April 15, 1998: Deadline hotel reservation**
**May 1, 1998: Abstract notification**
**June 1, 1998: Deadline late registration**
**June 16-19, 1998: 10th NCI-EORTC Symposium on New Drugs in Cancer Therapy**

More information for instance on abstract sumission or registration can be obtained from

**Dr. M. R. L. Krul**
**Conference Coordinator**
**EORTC New Drus Development Office**
**P. O. Box 7057**
**NL- 1007 MB Amsterdam, The Netherlands**

**Phone: + 31 20 444 2768**
**Fax: + 31 20 444 2699**
**e-mail: krul@euronet.nl**

Additional information can also be found on the home page oif the Symposium at
**http://www.eortc.be/seminar/newdrugs**

